# A lack of sexual autonomy is associated with increased loneliness in young mothers

**DOI:** 10.1186/s12905-024-03479-0

**Published:** 2025-01-04

**Authors:** Chelsea Bunke, Tara Keck

**Affiliations:** Department of Neuroscience, Physiology and Pharmacology, University College London, 21 University Street, London, WC1E 6DE UK

**Keywords:** Loneliness, Mothers, Gender, Sexual autonomy, The Republic of Moldova

## Abstract

**Background:**

Loneliness is a significant risk factor for both mental and physical health issues, including depression and increased mortality. Loneliness is reported at higher levels during life transitions, such as the transition to motherhood. Loneliness in mothers has far-reaching detrimental impacts on both mother and child, such as an increased risk of maternal depression and child abuse. Understanding the impact of different risk factors for loneliness, specifically in young mothers, may inform potential interventions for this at-risk group. The aim of this study was to determine whether mothers were lonelier than childfree women, and whether there are different risk factors for loneliness in mothers relative to childfree women, both for gender-associated and established risk factors for loneliness.

**Methods:**

This cross-sectional study included partnered mothers and partnered childfree women between the ages of 20 and 29 from the 2020 Generations and Gender Survey (GGS) in the Republic of Moldova. The De Jong Gierveld Loneliness Scale was used to assess overall, emotional, and social loneliness. A total of 11 potential risk factors were considered, across gender, well-being, relationships, and household status. Depending on the nature of the variables and their distributions, Wilcoxon rank-sum tests or Spearman correlation coefficients were used to assess loneliness risk factors for partnered mothers and childfree women.

**Results:**

Data from 396 mothers and 113 childfree women in the Republic of Moldova were analysed in this study. There was no significant difference between the mean overall, emotional, or social loneliness scores in partnered mothers and childfree women. A lack of sexual autonomy was a risk factor associated with social loneliness in young mothers, but not in childfree women. This was the only gendered risk factor that differed between populations. Other gendered risk factors were not significant for any types of loneliness in either population. There were differences between mothers and childfree women in several established risk factors for loneliness.

**Conclusion:**

Mothers were not lonelier than childfree women in this study, but a lack of sexual autonomy was a risk factor associated with loneliness only in mothers.

## Background

Loneliness is the difference between a person’s desired and actual social relationships [1] and is prevalent globally [2]. Chronic loneliness is associated with a number of health concerns, including an increased risk for heart disease [3], increased mortality [4], and increased depressive symptoms [5].

While loneliness is prevalent throughout society, comparative studies have shown that some populations may have a higher risk for loneliness and different populations may have different risk factors for loneliness [6–9]. For example, the risk of loneliness increases during life transitions [10], with many studies focusing on populations of older people [11, 12] who may experience many transitions in later life, such as bereavement, retirement, and physical changes. There are indications that the transition to motherhood, particularly in young women, is also a period where there is an increased risk of loneliness [13, 14]. Mothers experience changes in the body, as well as a disruption to social networks [15], a strain on relationships [16], new responsibilities [17], and a change in self-image [18]. This intense transition may leave mothers susceptible to loneliness, which is associated increased rates of depression in mothers and worse outcomes for children [19–22]; however, as risk factors for loneliness vary across the lifespan [23], the risk factors for loneliness that mothers experience may be different from those for older people or the general population. Identifying these risk factors associated with loneliness in mothers is key to develop interventions specifically for this group.

Loneliness is commonly separated into two components: social loneliness, having a lower quantity of relationships or smaller social network than desired, and emotional loneliness, not having enough high-quality relationships [15]. These two types of loneliness are often correlated but can have different risk factors. Separating these distinct aspects of loneliness is key, as there are specific health-related risks associated with each type of loneliness. Further, the risk factors associated with each type can be overlooked when only examining overall loneliness [24] and identifying these risk factors is critical for developing effective interventions.

Gendered risk factors, such as a perceived lack of gender equality in relationships and intimate partner violence (IPV), including a lack of sexual autonomy (e.g. not having the ability to say no to intercourse with a partner), have been indirectly implicated as risk factors for loneliness in mothers. For example, gender roles can be reinforced by the arrival of a child [25], particularly where the mother takes on a role as the primary unpaid household labourer. In a 2023 review of qualitative studies of perinatal depression and isolation in mothers, a gender dynamic in which the mother was responsible for a majority of childcare was found to be a risk factor for feeling isolated [16], which is often associated with loneliness. Furthermore, postpartum IPV has been shown to contribute to depression in mothers [26], which is also closely associated with loneliness. But to date, the relationship between gendered risk factors and loneliness in mothers is largely unclear.

Additionally, numerous risk factors for all types of loneliness have been identified for women and in some instances specifically mothers. Risk factors related to well-being, relationships, and household status have been shown to be relevant. These include depressive symptoms [5], life satisfaction [27], poor relationships with partners [14], and having a child with a disability [28]. Furthermore, a lack of internet-based communication [29], not having sufficient help with household tasks [30], lower education levels [31], a lack of work-life balance [32], and financial instability [30, 33] have all been demonstrated to be risk factors for loneliness and may affect mothers preferentially.

This study aims to examine loneliness levels in mothers, as well as gendered and established risk factors associated with loneliness for mothers. This study tests three main hypotheses:

1. Partnered young mothers are lonelier than partnered childfree women.
2. Gendered risk factors are associated with loneliness in partnered young mothers, but not partnered childfree women.
3. Established risk factors for loneliness are not different between partnered young mothers and partnered childfree women.

## Methods

### Data

To understand 1) the degree to which mothers were lonely relative to childfree women and 2) the specific risk factors for loneliness in mothers and childfree women, cross-sectional demographic data from the Generations and Gender Survey (GGS) [34] in the Republic of Moldova were analysed. This survey included 10,044 participants aged 15 to 79 and ran from January to December 2020. The analysis in this study used data from women aged 20 to 29 in the Republic of Moldova. Responses from 396 mothers and 113 childfree women were analysed. Respondents who did not answer the questions related to loneliness were excluded, as were women who did not have a partner. The Republic of Moldova was selected as the country of analysis in this study because the higher levels of IPV [35] may make gendered risk factors particularly relevant and the number of responses to the gendered variables were sufficient for analysis.

### Measures

Loneliness was assessed using the 6-item De Jong Gierveld scale [36, 37], which has been validated for populations of this demographic, specifically in the context of the GGS [37]. The scale consists of three questions each for social and emotional loneliness. For social loneliness, participants were asked to what extent these statements had applied to them recently: there are plenty of people I can rely on when I have problems**,** there are many people I can trust completely, and there are enough people that I feel close to**.** For emotional loneliness, the statements presented to participants were: I experience a general sense of emptiness, I miss having people around, and often, I feel rejected [34]. Both emotional and social loneliness were quantified, with scores ranging from 0 (not lonely) to 3 (extremely lonely). To measure overall loneliness, the responses were summed across social and emotional loneliness with overall loneliness scores ranging from 0 (not lonely) to 6 (extremely lonely).

Potential risk factors considered in this study were selected based on previous studies of loneliness in the general population, with a focus on risk factors that may particularly affect gender and motherhood. Questions related to gender attitudes [38] and sexual autonomy [39] were included in the study as the gendered variables. Well-being variables included depressive symptoms [5] and life satisfaction [27]. Partner relationship satisfaction was included as a risk factor for relationships [14]. Having a child with a disability was only included for mothers [28] as a risk factor linked to relationships. Household variables included having an internet connection [29], satisfaction with household tasks [30], education level [31], work-life balance [32], and financial stability [30, 33].

The sexual autonomy variable was defined as having the ability to say no to the respondent’s partner if the respondent did not want to have intercourse [34], with respondents answering either yes or no to whether they have this ability. Questions related to gender attitudes included five questions, originally based on the World Values Survey [40]. Participants were asked whether men or women were better political leaders, for whom university is most important, for whom having a job is most important, for whom looking after a child is more important, and whether men or women were better at childcare [34]. The scores for the responses to these questions were combined, with the final ‘questions related to gender attitudes’ score ranging from 0 to 5, with lower values reflecting more traditional gender ideals.

Depressive symptoms were measured by the Center for Epidemiological Studies Depression (CESD) five factor scale [34, 41]. Respondents were asked how often they felt like they couldn’t shake off the blues, felt depressed, felt like a failure, felt fearful, and felt sad, with answers on a scale from 1 to 4. A value of 1 for a given response meant they never felt that way, and a value of 4 indicated that they felt that way most or all of the time. The mean score across the five questions was used in this study to minimize the effects of missing responses. Therefore, the scores ranged from 1 to 4, with 1 indicating low depressive symptoms and 4 indicating high depressive symptoms. Life satisfaction, partner relationship satisfaction, and household task satisfaction were reported on a scale from 0 to 10, with higher values reflecting higher levels of satisfaction. Education scores ranged from 1 to 9, as reported using the International Standard Classification of Education (ISCED), with the lowest score (1) reflecting less than primary education, and the highest score (9) reflecting a doctoral or equivalent education. Work-life balance was constructed based on responses to four questions: respondents were asked how often they felt too tired to do chores, too tired to function at work, that it was difficult to concentrate because of family responsibilities, and that it was difficult to fulfil family responsibilities. Scores of 1 reflected that respondents felt this way several times a week, and scores of 4 indicated that respondents never felt this way. Therefore, higher values reflect more work-life balance. The mean score across these variables was recorded for each respondent. It is important to note that only employed respondents answered this question. Financial stability was calculated by summing the responses to the following questions: can you make ends meet, could you not pay bills in the last month, and can your household afford 11 types of household expenses: keeping the house warm, taking a week of holiday, replacing furniture, buying new clothes, eating meat, entertaining friends and family, managing unexpected expenses, access to a car, owning two pairs of shoes, having pocket money, and leisure activities. This variable was constructed such that higher values reflect more financial stability, with a minimum of 0 and a maximum of 17.

### Statistics

The primary outcomes of the tests were overall, emotional, and social loneliness scores, as measured by the De Jong Gierveld scale. To assess whether there was a difference in loneliness score distributions between partnered mothers and childfree women, Wilcoxon-rank sum tests were conducted to compare the overall, emotional, and social loneliness score distributions of the two populations. For risk factor variables, mothers and childfree women were analysed separately. Wilcoxon rank-sum tests with Bonferroni-Holm corrections [42] were conducted to compare the overall, emotional, and social loneliness score distributions between respondents answering yes/no to the binary variables. For scaled variables, correlations with overall, emotional, and social loneliness scores were measured using Spearman’s rank correlation coefficient, and Bonferroni-Holm adjusted p-values were reported [42]. For all measures, an adjusted p-value of less than 0.05 was considered significant. In some instances, subsampling was conducted on the sample of mothers to ensure that mothers and childfree women with the same mean age were being analysed and that significant results were consistent when controlled for age. There were no observed differences between subsampled results and the results from the entire population, so results from the entire population have been reported.

## Results

Overall, emotional, and social loneliness scores in partnered mothers and childfree women are reported in Table 1. No significant differences in overall, emotional, or social loneliness scores were found between the two populations. Risk factors for loneliness were assessed separately for partnered mothers (Tables 2 and 4) and partnered childfree women (Tables 3 and 5), with a focus on gendered risk factors (Tables 2 and 3) and established risk factors (Tables 4 and 5).

**Table 1:** Mean loneliness scores in partnered mothers and childfree women. Overall loneliness scores range from 0 (not lonely) to 6 (extremely lonely). Emotional and social loneliness scores range from 0 (not lonely) to 3 (extremely lonely). Comparisons between loneliness scores of partnered mothers and childfree women were made using a Wilcoxon rank-sum test. SD, Standard deviation. n, the total number of respondents who answered the prompt

	**Mean Loneliness (SD)**
**Overall Loneliness**	
Mothers (*n* = 392)	2.054 (1.481)
Childfree women (*n* = 111)	1.928 (1.500)
	*p* = 0.252
**Emotional Loneliness**	
Mothers (*n* = 396)	1.202 (0.874)
Childfree women (*n* = 112)	1.125 (0.818)
	*p* = 0.371
**Social Loneliness**	
Mothers (*n* = 393)	0.858 (1.071)
Childfree women (*n* = 113)	0.814 (1.082)
	*p* = 0.588

**Table 2:** Gendered risk factors associated with loneliness in partnered mothers. Overall loneliness ranges from 0 (not lonely) to 6 (extremely lonely) while emotional and social loneliness scores range from 0 (not lonely) to 3 (extremely lonely). For each binary variable, comparisons are made using a Wilcoxon rank-sum test between loneliness scores of respondents answering yes or no. Bonferroni-Holm-adjusted *p*-values are reported. For all other variables, correlations are measured between the variable and loneliness scores. Spearman correlation coefficients and Bonferroni-Holm-adjusted *p*-values are reported. SD, Standard deviation. IQR, Interquartile range. n, the total number of respondents who answered the prompt. r, the Spearman correlation coefficient. Bolded *p*-values indicate significance (*p* < 0.05)

	**Overall Loneliness**	**Emotional Loneliness**	**Social Loneliness**
	Percent yes Mean loneliness (SD)	Percent yes Mean loneliness (SD)	Percent yes Mean loneliness (SD)
**Gender**			
Sexual autonomy	83.3% (*n* = 365)	83.5% (*n* = 369)	83.3% (*n* = 366)
Yes	1.984 (1.490)	1.211 (0.851)	0.780 (1.053)
No	2.344 (1.525)	1.115 (0.943)	1.230 (1.122)
	*p* = 0.442	*p* = 1.000	***p* = 0.010**
Variable (range) Median [IQR]			
**Gender**			
Questions related to gender attitudes (0-5) 4.0 [3.0 4.0]	*r* = 0.005 (*n* = 388) * p* = 1.000	*r* = 0.078 (*n* = 391) * p* = 0.364	*r* = -0.058 (*n* = 388) * p* = 0.510

**Table 3:** Gendered risk factors associated with loneliness in partnered childfree women. Overall loneliness ranges from 0 (not lonely) to 6 (extremely lonely) while emotional and social loneliness scores range from 0 (not lonely) to 3 (extremely lonely). For each binary variable, comparisons are made using a Wilcoxon rank-sum test between loneliness scores of respondents answering yes or no. Bonferroni-Holm-adjusted *p*-values are reported. For all other variables, correlations are measured between the variable and loneliness scores. Spearman correlation coefficients and Bonferroni-Holm-adjusted *p*-values are reported. SD, Standard deviation. IQR, Interquartile range. n, the total number of respondents who answered the prompt. r, the Spearman correlation coefficient. Bolded *p*-values indicate significance (*p* < 0.05)

	**Overall Loneliness**	**Emotional Loneliness**	**Social Loneliness**
	Percent yes Mean loneliness (SD)	Percent yes Mean loneliness (SD)	Percent yes Mean loneliness (SD)
**Gender**			
Sexual autonomy	81.8% (*n* = 99)	82.0% (*n* = 100)	82.0% (*n* = 100)
Yes	1.963 (1.551)	1.171 (0.853)	0.805 (1.109)
No	2.000 (1.599)	1.222 (0.711)	0.778 (1.133)
	*p* = 1.000	*p* = 1.000	*p* = 1.000
Variable (range) Median [IQR]			
**Gender**			
Questions related to gender attitudes (0-5) 4.0 [3.0 4.0]	*r* = -0.082 (*n* = 108) * p* = 0.795	*r* = -0.131 (*n* = 108) * p* = 1.000	*r* = -0.020 (*n* = 110) * p* = 1.000

**Table 4:** Well-being, relationship, and household risk factors associated with loneliness in partnered mothers. Overall loneliness ranges from 0 (not lonely) to 6 (extremely lonely) while emotional and social loneliness scores range from 0 (not lonely) to 3 (extremely lonely). For each binary variable, comparisons are made using a Wilcoxon rank-sum test between loneliness scores of respondents answering yes or no. Bonferroni-Holm-adjusted *p*-values are reported. For all other variables, correlations are measured between the variable and loneliness scores. Spearman correlation coefficients and Bonferroni-Holm-adjusted *p*-values are reported. SD, Standard deviation. IQR, Interquartile range. n, the total number of respondents who answered the prompt. r, the Spearman correlation coefficient. Bolded *p*-values indicate significance (*p* < 0.05)

	**Overall Loneliness**	**Emotional Loneliness**	**Social Loneliness**
	Percent yes Mean loneliness (SD)	Percent yes Mean loneliness (SD)	Percent yes Mean loneliness (SD)
**Relationships**			
Child has a disability	5.1% (*n* = 392)	5.3% (*n* = 396)	5.1% (*n* = 393)
Yes	2.200 (1.327)	1.190 (0.906)	1.000 (0.949)
No	2.046 (1.487)	1.203 (0.871)	0.850 (1.076)
	*p* = 1.000	*p* = 1.000	*p* = 0.868
**Household**			
Internet connection	82.7% (*n* = 392)	82.6% (*n* = 396)	82.4% (*n* = 393)
Yes	2.009 (1.481)	1.199 (0.864)	0.809 (1.051)
No	2.265 (1.451)	1.217 (0.915)	1.087 (1.126)
	*p* = 0.913	*p* = 1.000	*p* = 0.273
Variable (range) Median [IQR]			
**Well-being**			
Depressive symptoms (1-4) 1.6 [1.2 1.8]	*r* = 0.400 (*n* = 392) ***p* < 0.001**	*r* = 0.378 (*n* = 396) ***p* < 0.001**	*r* = 0.266 (*n* = 392) ***p* < 0.001**
Life satisfaction (0-10) 9.0 [8.0 10.0]	*r* = -0.229 (*n* = 391) ***p* < 0.001**	*r* = -0.221 (*n* = 395) ***p* < 0.001**	*r* = -0.155 (*n* = 391) ***p* = 0.015**
**Relationships**			
Partner relationship satisfaction (0-10) 10.0 [9.0 10.0]	*r* = -0.162 (*n* = 392) ***p* = 0.005**	*r* = -0.117 (*n* = 396) *p* = 0.079	*r* = -0.122 (*n* = 393) ***p* = 0.047**
**Household**			
Household task satisfaction (0-10) 10.0 [9.0 10.0]	*r* = -0.159 (*n* = 326) ***p* = 0.012**	*r* = -0.069 (*n* = 330) *p* = 0.419	*r* = -0.152 (*n* = 326) ***p* = 0.030**
Education (1-9) 5.0 [4.0 7.0]	*r* = -0.184 (*n* = 392) ***p* = 0.001**	*r* = -0.139 (*n* = 396) ***p* = 0.033**	*r* = -0.137 (*n* = 393) ***p* = 0.030**
Work-life balance (1-4) 3.0 [2.25 3.75]	*r* = -0.063 (*n* = 73) *p* = 1.000	*r* = -0.019 (*n* = 74) *p* = 0.871	*r* = -0.046 (*n* = 73) *p* = 0.699
Financial stability (0-17) 10.0 [6.0 13.0]	*r* = -0.192 (*n* = 359) ***p* = 0.002**	*r* = -0.136 (*n* = 359) ***p* = 0.049**	*r* = -0.161 (*n* = 359) ***p* = 0.015**

**Table 5:** Well-being, relationship, and household risk factors associated with loneliness in partnered childfree women. Overall loneliness ranges from 0 (not lonely) to 6 (extremely lonely) while emotional and social loneliness scores range from 0 (not lonely) to 3 (extremely lonely). For each binary variable, comparisons are made using a Wilcoxon rank-sum test between loneliness scores of respondents answering yes or no. Bonferroni-Holm-adjusted *p*-values are reported. For all other variables, correlations are measured between the variable and loneliness scores. Spearman correlation coefficients and Bonferroni-Holm-adjusted *p*-values are reported. SD, Standard deviation. IQR, Interquartile range. n, the total number of respondents who answered the prompt. r, the Spearman correlation coefficient. Bolded *p*-values indicate significance (*p* < 0.05)

	Overall Loneliness	Emotional Loneliness	Social Loneliness
	Percent yes Mean loneliness (SD)	Percent yes Mean loneliness (SD)	Percent yes Mean loneliness (SD)
**Household**			
Internet connection	% yes = 82.9 (*n* = 111)	% yes = 82.1 (*n* = 112)	% yes = 82.3 (*n* = 113)
Yes	1.815 (1.459)	1.130 (0.783)	0.699 (1.045)
No	2.474 (1.535)	1.100 (0.788)	1.350 (1.062)
	*p* = 0.190	*p* = 1.000	***p* = 0.029**
Variable (range) Median [IQR]			
**Well-being**			
Depressive symptoms (1-4) 1.6 [1.2 1.8]	*r* = 0.417 (*n* = 111) ***p* < 0.001**	*r* = 0.352 (*n* = 112) ***p* = 0.001**	*r* = 0.326 (*n* = 113) ***p* = 0.003**
Life satisfaction (0-10) 9.0 [8.0 10.0]	*r* = -0.225 (*n* = 110) *p* = 0.095	*r* = -0.111 (*n* = 111) *p* = 0.584	*r* = -0.191 (*n* = 112) *p* = 0.260
**Relationships**			
Partner relationship satisfaction (0-10) 10.0 [9.0 10.0]	*r* = -0.228 (*n* = 111) *p* = 0.095	*r* = -0.163 (*n* = 112) *p* = 0.432	*r* = -0.187 (*n* = 113) *p* = 0.260
**Household**			
Household task satisfaction (0-10) 10.0 [8.5 10.0]	*r* = -0.067 (*n* = 67) *p* = 0.795	*r* = -0.246 (*n* = 67) *p* = 0.268	*r* = 0.032 (*n* = 68) *p* = 1.000
Education (1-9) 5.0 [4.0 6.0]	*r* = -0.132 (*n* = 111) *p* = 0.499	*r* = -0.123 (*n* = 112) *p* = 0.701	*r* = -0.075 (*n* = 113) *p* = 1.000
Work-life balance (1-4) 3.0 [2.5 3.5]	*r* = -0.354 (*n* = 60) ***p* = 0.039**	*r* = -0.269 (*n* = 60) *p* = 0.266	*r* = -0.368 (*n* = 61) ***p* = 0.025**
Financial stability (0-17) 11.0 [8.5 13.0]	*r* = -0.187 (*n* = 102) *p* = 0.421	*r* = -0.079 (*n* = 103) 1.000	*r* = -0.158 (*n* = 103) *p* = 0.779

### Gendered risk factors

The gendered risk factors associated with loneliness for the populations of partnered mothers (Table 2) and childfree women (Table 3) were assessed. One variable under the theme of gender had a significant association with loneliness: sexual autonomy, measured as the perceived ability to say no to sexual intercourse with a partner. Mothers who lacked sexual autonomy had significantly higher social loneliness scores than mothers who had sexual autonomy (Table 2); however, no other associations were found between gendered risk factors and any type of loneliness in mothers (Table 2). In childfree women, no gendered risk factors were associated with any type of loneliness (Table 3).

### Well-being risk factors

Well-being risk factors for loneliness were examined for partnered mothers (Table 4) and childfree women (Table 5). Depressive symptom scores positively correlated with overall, emotional, and social loneliness scores in both mothers (Table 4) and childfree women (Table 5). Life satisfaction inversely correlated with all types of loneliness in mothers (Table 4), but not in childfree women (Table 5).

### Relationship risk factors

Relationship risk factors for loneliness were examined for partnered mothers (Table 4) and childfree women (Table 5). Partner relationship satisfaction was inversely correlated with overall and social loneliness in mothers (Table 4) but was not significantly associated with any type of loneliness in childfree women (Table 5). Having a child with a disability was not associated with any type of loneliness in mothers (Table 4).

### Household risk factors

Household risk factors for loneliness were examined for partnered mothers (Table 4) and childfree women (Table 5). Childfree women who did not have an internet connection had higher social loneliness scores than childfree women who did (Table 5), but not having an internet connection was not associated with any type of loneliness in mothers (Table 4). Household task satisfaction was inversely correlated with overall and social loneliness in mothers (Table 4) but was not associated with any type of loneliness in childfree women (Table 5). Education levels were inversely correlated with overall, emotional, and social loneliness in mothers (Tables 4), and no types of loneliness in childfree women (Table 5). Work-life balance was inversely correlated with overall and social loneliness in childfree women (Table 5), but not with any type of loneliness in mothers (Table 4) (74 out of 396 mothers and 61 out of 113 childfree women reported being employed). Finally, financial stability was inversely correlated with overall, emotional, and social loneliness in mothers (Table 4), such that higher levels of financial stability were associated with lower loneliness scores, but not with any type of loneliness in childfree women (Table 5).

## Discussion

This study had three hypotheses: 1) partnered young mothers are significantly lonelier than partnered childfree women, 2) gendered risk factors are associated with loneliness in partnered young mothers, but not partnered childfree women, and 3) established risk factors for loneliness are not different between partnered young mothers and partnered childfree women. The first hypothesis was not supported. This study found no significant differences between loneliness scores in mothers and childfree women aged 20-29. The second hypothesis was supported. Mothers without sexual autonomy had significantly higher social loneliness scores than mothers with sexual autonomy, and this risk factor was not significant for childfree women. The third hypothesis was not supported. There were differences in risk factors between partnered mothers and childfree women, with life satisfaction, partner relationship satisfaction, household task satisfaction, education, and financial stability being risk factors for mothers, but not childfree women, and internet connection and work life balance being risk factors for childfree women, but not mothers.

### Mothers are not lonelier than childfree women in the Republic of Moldova

Across overall, emotional, and social loneliness scores, there were no significant differences between partnered mothers and childfree women. While previous studies have not directly compared loneliness levels between mothers and age-matched childfree women, Rokach [43] measured self-alienation, interpersonal isolation, social inadequacy, and emotional distress, all of which may influence loneliness levels, in populations of Canadian women. They found that, compared with women from the general population, mothers reported significantly higher levels for all of these measures. Thus, while loneliness levels may not diverge significantly between childfree women and mothers, the findings of Rokach [43] and the risk factor analysis from this study in the Republic of Moldova support the notion that the risk factors for loneliness may differ between these populations.

### Risk factors for loneliness

The results of this study indicate that a lack of sexual autonomy is associated with higher social loneliness in mothers. On average, mothers without sexual autonomy had social loneliness scores 0.45 points higher on a three-point scale than mothers with sexual autonomy (15% increase). To provide context for the magnitude of this difference, a recent study on the impact of COVID-19 lockdowns on loneliness in young people found that three months of lockdown was associated with a 0.5-point increase on a six-point overall loneliness scale (8% increase) [44]. While it is difficult to make precise comparisons across studies with differing methodologies, this suggests that the increase in loneliness associated with a lack of sexual autonomy in mothers may be an important public health concern.

While this is the first study to examine sexual autonomy, a form of IPV, as a risk factor for loneliness in mothers, past studies have suggested a relationship between IPV and loneliness. In a study from the United States, women with past experiences of IPV (psychological, physical, and sexual) were more likely to report loneliness, and lonely women were more likely to find themselves in IPV relationships in the future [45]. The Republic of Moldova has one of the higher reported rates of IPV in Europe (14.6%) [35], indicating that a sizeable subset of women may be affected. Previous studies in Asia and Europe suggest that societies with higher levels of IPV also have higher levels of stigma surrounding reporting [46, 47], which may lead to further social isolation and loneliness. Further, women demonstrating consensual sexual engagement that they do not desire (sexual compliance), which is a potential manifestation of a lack of sexual autonomy [48], reported negative consequences on their well-being [49]. While these results are aligned with the findings in this study, no studies have been conducted on sexual compliance and loneliness to date.

A lack of sexual autonomy may differentially impact mothers given the importance of intimate relationships reported in this population. In Lee et al. [14], mothers in the UK reported that issues in their intimate relationships had a larger impact on their well-being upon entering motherhood. Therefore, when mothers feel sexually powerless in intimate relationships, it could exacerbate feelings of loneliness. Consistent with Lee et al. [14], the results in this study show that low partner relationship satisfaction was associated with higher scores of overall and social loneliness in mothers, but partner relationship satisfaction was not significantly associated with any type of loneliness in childfree women. This idea is also consistent with previous work from Japan [50] that showed that low levels of partner support was associated with higher levels of loneliness in mothers.

Questions related to gender attitudes did not have a significant association with any type of loneliness in either population. Thorsteinsen et al. [51] showed that traditional gender ideologies about domestic care in mothers (i.e. the mother is a better caregiver) were associated with lower maternal well-being. A possible interpretation is that some aspects of well-being in mothers, such as depression and life satisfaction, are related to gender attitudes, but loneliness is less affected. Alternatively, the questions related to gender attitudes in this survey may not fully capture gender attitudes in women in the Republic of Moldova.

### Study limitations

The present study has some limitations. First, like many studies in the field of loneliness [24], it is difficult to determine the directionality of the relationship between loneliness and the identified risk factors. Previous work has suggested that for some risk factors, such as depression [52], the interactions with loneliness can be bidirectional, with positive feedback occurring between the two conditions. Additionally, health issues associated with loneliness tend to be associated with chronic loneliness [24], which cannot be quantified with the cross-sectional dataset used here. While acute loneliness can be an issue for health, it can also have a positive influence to motivate people to seek out social interactions and build more extensive social networks [23]. Future studies should aim to collect longitudinal data to disentangle the effects of chronic and acute loneliness. Finally, social participation is a key loneliness risk factor [53] but was not measured for populations under 65 in the GGS. Understanding if and how mothers’ social engagement may change could be informative for developing interventions to address loneliness in this group.

## Conclusions

The present study is the first to demonstrate that motherhood may be linked to different risk factors for loneliness compared to childfree women of the same age and to highlight the importance of gendered factors in loneliness in mothers. This study demonstrated that the gendered risk factor, a lack of sexual autonomy, is associated with social loneliness in mothers, but not in childfree women. Motherhood, its stressors and the surrounding social changes may put this population at a greater risk of loneliness when their sexual autonomy is compromised; however, no other gendered variables showed a significant association with any type of loneliness. This study further established that many common risk factors for loneliness in the general population are also risk factors for mothers. As loneliness in mothers is associated with increased rates of maternal depression and worse outcomes for children [19–22], understanding the risk factors for loneliness in this group is critical and can be used for informing interventions to reduce loneliness.

## Data Availability

The datasets generated and/or analysed during the current study are available in the Colectica repository, https://ggp.colectica.org/data/int.ggp/76c9f079-c4cb-4707-a3b1-808bf82a81ab/int.ggp/051cb995-166c-4c3c-8f62-790926f3e1a1/ined/8cbe66a3-ab58-472b-b745-8c0173214dc2 .

## Abbreviations

GGS: Generations and Gender Survey
IPV: Intimate Partner Violence
SD: Standard Deviation
IQR: Interquartile Range
n: Total number of respondents
r: Spearman correlation coefficient
